# Rapidly diverging public trust in science in the United States

**DOI:** 10.1177/09636625241302970

**Published:** 2024-12-07

**Authors:** Manjana Milkoreit, E. Keith Smith

**Affiliations:** University of Oslo, Norway; ETH Zurich, Switzerland

**Keywords:** ideology, partisanship, science, social tipping point, trust, United States

## Abstract

Trust in science is crucial to resolving societal problems. Americans across political ideologies have high levels of trust in science—a stable pattern observed over the past 50 years. Yet, trust in science varies by individual and group characteristics and faces several threats, from political actors, increased political polarization, or global crises. We revisit historical trends of trust in science among Americans by political orientation. We find steadily diverging trends by political views since the 1990s, and a drastically and rapidly opening gap since 2018. Recent unprecedented changes are driven not only by decreases in trust among conservatives but also by increases among liberals. Existing theoretical accounts do not fully explain these patterns. Diverging attitudes toward the institution of science can diminish capacity for collective problem-solving, eroding the shared foundation for decision-making and political discourse.

## 1. Public trust in science—A bedrock of society

Public trust in science is fundamental to a well-functioning society. Scientific knowledge generation serves to identify and understand societal problems, to develop social and technological solutions, to inform public policy (Resnik, 2011), and to foster innovation. Public trust in scientific institutions is a crucial element for resolving collective action problems, such as following scientifically informed behavioral recommendations for mask-wearing during a pandemic (Hamilton and Safford, 2021; Harring et al., 2021). Mistrust in science, on the other hand, hampers a society’s ability to address collective problems and erodes shared understandings of reality—the very foundation of social life and political systems.

Trust in science refers to popular perceptions of science as a cultural institution with a distinct role in society and relationship to the state (Gauchat, 2015, 2023). One can distinguish trust in individual or groups of scientists, trust in scientific methods and principles, and trust in the institutions of science (e.g. Achterberg et al., 2017), that is, the organized networks and organizations bound by norms and standardized processes of decision-making. Here, we use the terms trust or confidence in science to refer to this latter institutional understanding of science.

**Figure 1.**
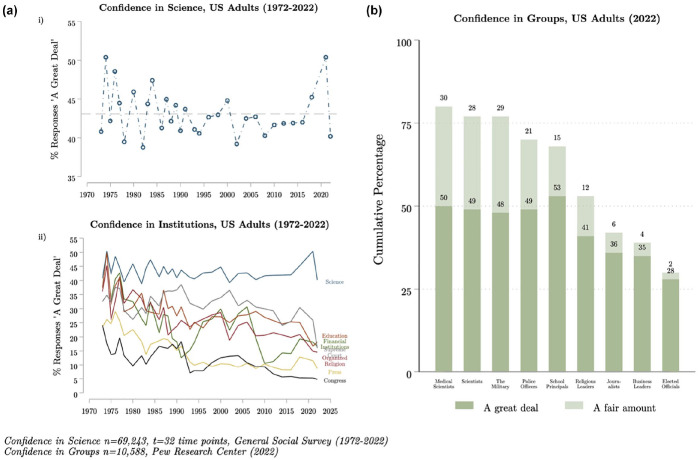
Confidence in science among Americans. Panel A[i] displayed the percentage of Americans with “a great deal” of confidence in science, from 1972 to 2022. Panel A[ii] presents the percentage of Americans with “a great deal” of across societal institutions (science, education, financial institutions, the Supreme Court, organized religion, press, and congress) from 1972 to 2022. Panel B shows the percentage of Americans with “a great deal” and “a fair amount” of confidence across social groups (medical scientists, scientists, the military, political officers, school principals, religious leaders, journalists, business leaders, elected officials) in 2022. The data for Panel A come from the General Social Survey (1972–2022, *n* = 62,243, *t* = 32 time points); the data for Panel B come from the Pew Research Center, American Trends Panel (2022 (*n* = 10,588)).

Over previous decades, trust in science in the United States has remained stable (Krause et al., 2019) at comparatively higher levels than trust in other institutions and societal groups (Figure 1, Panel A), and it has been rather resistant against individual events or negative information (Siegrist, 2021). Changes in levels of trust have usually remained limited to specific issues or segments of society (e.g. conservative attitudes toward climate change science), have occurred rather slowly, and been of limited duration. While recent studies observed a decline in Americans trust in science over the last few years (Lupia et al., 2024), it has remained greater than trust in representatives of many other prominent institutions, including the military, police, teaching, and religion (Figure 1, Panel B).

Yet, trust in science varies substantially by individual and group characteristics within American society. Various theories seek to explain these differences, for example, with norm following and behavioral alignment within social identity groups (Fielding and Hornsey, 2016) or the role of elite cues (Hamilton and Safford, 2021). There is strong evidence that political ideology rather than facts condition trust in climate science (Gauchat, 2012; Mooney, 2006), including loss of trust (Leiserowitz et al., 2010), and that the growing role of political ideology as social identities has been contributing to the polarization of views on science (and other issues) in the United States (Cramer, 2016; Mason, 2018).

Challenges to ideology-based explanations (Nisbet et al., 2015) have opened a more nuanced debate, for example, identifying specific values and attitudes embedded within an ideology or different strands of an ideology (e.g. Gauchat, 2015) as determinants of trust (McCright et al., 2013; Pechar et al., 2018) or exploring issue-specific differences in trust (Hamilton, 2015; McCright et al., 2013). This recent scholarship has moved toward explanations based on the specific characteristics of policy issues (e.g. climate change, health, genetically modified food). The emerging, more complicated understanding suggests that trust is a function of the relationship between political values, issue-specific scientific knowledge, and its implications for policy-making (Pechar et al., 2018). When presented with scientific information about a policy that threatens a person’s political values (Nisbet et al., 2015), they are likely to express doubts about the scientific facts and lower levels of trust in science, as a defense against this value threat. This dynamic of motivated reasoning affects liberals and conservatives alike. Based on these advances, differences in trust between conservatives and liberals can only be understood when looking at the relationships between a specific ideology and the social-political issue at hand. However, explanations that rely on ideology-issue linkages so far fail to address changes in general trust in science. Less is known about whether and how sub-group dynamics shape general (i.e. issue-independent) levels of public trust in science over time.

Here, we ask whether the role of science in society itself—its status as a cultural institution—has become a politicized issue, where individuals’ levels of trust in science depend upon their ideology. We investigate decades-long dynamics of trust in science in the United States from 1972 to 2022, exploring particularly differences between ideological groups. We find a recent, surprisingly rapid, and significant shift in patterns of general trust in science among Americans, generated by decreased levels of trust among political conservatives paired with simultaneous increases among liberals. These substantive shifts of trust in science exceed the decades-long boundaries of variability, suggesting a potential paradigm shift toward sustained, heightened polarization of Americans’ attitudes toward science. We discuss the implications of this observation, and the future research questions it raises.

## 2. Research design and methods

**Figure 2.**
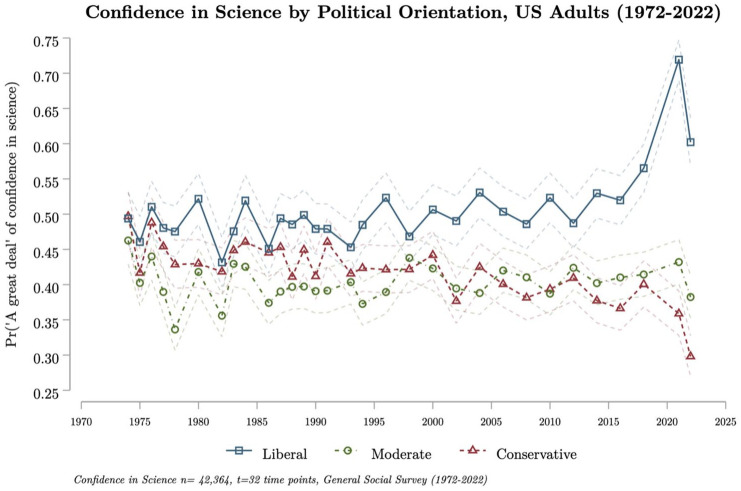
Confidence in science among Americans by political orientation, 1972–2022. The average predicted probability of having “a great deal” of confidence in science, by political orientation (liberal, moderate, conservative) in the United States from 1972 to 2022. The average predicted probability is calculated from CCREM estimates of confidence in science by yearly effects, adjusting for the respondent age and birth year cohort, and controlling for gender, ethnic identities, and education. The 95% confidence interval for the average predicted probability is plotted in dashed line. The data come from the General Social Survey (1972-2022, *n* = 42,364, *t* = 32 time points).

Here, we draw upon two different sources of data. Both are secondary survey data that directly inquire into respondents’ trust or confidence in the institution of science rather than discrete indicators of trustworthiness. While doubts have been raised about the usefulness of direct measures (Besley and Tiffany, 2023), these are the only ones currently available for such longitudinal analyses. First, we use pooled cross-sectional survey data from the General Social Survey (GSS) to explore trends in trust in science in the United States from 1972 to 2022 (*n* = 42,364, *t* = 32) among all Americans (Figure 1, Panel A(i)) and by political orientation (Figure 2).

The dependent variable *trust in science* is operationalized using the following item:

I am going to name some institutions in this country. As far as the people running these institutions are concerned, would you say you have a great deal of confidence, only some confidence, or hardly any confidence at all in them?: Scientific Community.

Response options include (1) a great deal, (2) only some, (3) hardly any. Here, *trust in science* for all Americans is identified as the percentage of people having “a great deal” of trust in science for each available survey year. The independent variable political orientation is operationalized via a question asking respondents to rate their political views on a seven-item scale ranging from extremely liberal to extremely conservative. For parsimony and to ease substantive interpretation, we recode this variable into a three-item scale (liberal, moderate, and conservative). This approach is similar to previous operationalizations of political orientation for studies of scientific trust in the United States (e.g. Gauchat, 2012; McCright et al., 2013; Nisbet et al., 2015).

We adopted a regression-based technique suited for historical cross-sectional survey data (CCREM). The CCREM approach estimates trust in science by yearly effects (period), while adjusting for the respondent age and birth year cohort (Yang, 2008; Yang and Land, 2013). Here, we allow for political orientation to vary by survey year period (random effects), controlling gender and ethnic identities, as well as level of education. To enable substantive interpretation of CCREM estimates, we calculate the predicted probability of having “a great deal” of trust in science for each year, by political orientation in Figure 1, Panel B. The predicted probabilities can be interpreted as the likelihood of a respondent with liberal, moderate, or conservative political views having “a great deal” of confidence in science for any given survey year.

To calculate trust in different societal institutions, we draw upon a second set of items from the GSS, utilizing perceptions of confidence in the scientific community (“science”), banks and financial institutions (“financial institutions”), “organized religion,” “education “press,” US supreme court (“supreme court”), and “congress.” These items are all originally captured with a response scale ranging from 1 = “a great deal,” 2 = “only some,” and 3 = “hardly any.” For our analyses, we similarly collapse these responses into a binary variable, 1 = “a great deal” and 0 = “not a great deal.” Figure 1A(ii) presents the average (mean) of having a great deal of confidence in each of these groups from 1972 to 2022 (*t* = 32 time points, *n* = 69,253 responses). The regression analyses control for the effects of gender, ethnic identities, and education.

Second, we use data from the Pew Research Center’s American Trends Panel (Pew Research Center, 2022) to identify contemporary confidence of Americans in different societal groups ranging from scientists to school principals to elected officials (*n* = 10,588). The Pew Research surveyed (*n* = 10,588) in September 2022 as part of the American Trends Panel. The data is a nationally representative, probability sample of persons living in the United States (18+). The survey asked confidence in several different societal groups using the question, “How much confidence, if any, do you have in each of the following to act in the best interests of the public?” The responses ranged from (1) no confidence at all to (4) a great deal of confidence. We display the cumulative percentage of Americans having either “a great deal” or “a fair amount” of confidence in Figure 1, Panel A(ii).

All analyses were performed using Stata 16.1.

## 3. Long-term divergence opening a massive gap

Americans have been consistently very trusting in science over the past 50 years. From 1972 to 2022, between 40% and 50% of Americans have had “a great deal” of confidence in science (Figure 1, Panel A(i)). Trust in science has remained remarkably stable over this time, particularly in comparison with other societal institutions (Figure 1, Panel A(ii)). According to a Pew Research study from 2022 (Figure 1, Panel A(ii)), 77% of Americans have either a great deal or fair amount of trust in scientists—exhibiting substantively higher trust than police officers (70%), religious leaders (53%), or elected officials (30%), for example.

Historically, the differences in trust in science by political orientation have been rather small, and changes in both sub-groups followed similar patterns, where in both groups, trust increases (or decreases) at the same time.

Starting in the 1990s, political trust dynamics became less coupled, marking the beginning of a long-term diverging trend. The share of conservatives with a great deal of trust in science began to decrease, while the share of liberals steadily increased. Yet, until 2018, these levels of trust by political orientation had largely stayed within a bounded range, with each sub-group remaining within the historically established variability range for all Americans of ~35%–55%.

Yet, more recently, trust in science between liberal and conservatives has fully decoupled—opening an unprecedented gap. In 2022, liberals are twice as likely to have a great deal of trust in science than conservatives (predicted probability of 0.60 vs 0.30, respectively). This difference is the result of an upward shift in trust within liberals since 2016 (increasing from 0.52 to 0.60), and a similar decline of scientific trust among conservatives during the same time (decreasing from 0.37 to 0.30). Notably, there was a decrease in trust in science among liberals between 2021 (0.72) and 2022 (0.60). Yet, in 2022, liberals still had the second highest level of trust in science observed over the entire time period, a value exceeding historical boundaries.

A massive and still widening gap has opened in general public attitudes toward science in the United States. In 2021 and 2022, conservatives and liberals had the lowest and highest observed average levels of trust in science, respectively (see Figure A.1 in the Supplemental Information (SI)). Taken together, these findings signal a substantive and significant shift in Americans’ trust in science, an area of public perceptions that has so far remained robust against a general trend of deteriorating trust in societal institutions.

Finally, given the increasing correlation of political orientation and party affiliation in the United States over this time period (see Figure A.2 in the SI), we conducted robustness checks for these findings, utilizing a three-item operationalization of party affiliation (Republican, Independent, Democrat) in place of political orientation (see Figure A.3 in the SI). Furthermore, we estimated the models for political orientation using frequency of attendance in religious services as a control variable, yielding non-substantive differences in the results (see Figure A.4 in the SI). Finally, we adopt a CCREM-based approach to estimate the effect of changing levels of trust over time by political orientation, fitting with same methodological approach by several recent studies using the historical GSS dataset (Clark et al., 2020; Johnson and Schwadel, 2019; Smith et al., 2024). But, given the age, period, cohort identification problem, such findings are sensitive to estimation strategy. As a robustness check, we further estimated the same models using a fixed-effects-based approach, finding substantively similar results (see Figure A.5 in the SI). Accordingly, these supplementary analyses suggest robustness in these findings across operationalizations of political views and affiliation and estimation strategies.

## 4. Causes, duration, and implications

These observations raise several questions. First, and most importantly, what explains the increasing gap of trust in science between liberal and conservative voters beyond the previously observed gradual divergence since the 1990s (Gauchat, 2012)? Second, is the recent step change a temporary, reversible phenomenon, or are we observing a tipping process, that is, a rapid reorganization of the relationship between science and American society toward a new stable state? Third, what are the immediate and long-term implications of diminished trust in science? We address each of these questions in turn, scoping a new future research agenda within this mature field of social science scholarship.

### Potential explanations

Making sense of the sudden and massive divergence between trust in science among liberals and conservatives is not possible without considering the role of the presidency, political ideology, and party affiliation (Barber and Pope, 2019), and historical trends of heightened political polarization over the last three decades (Johnson and Schwadel, 2019). While these findings support arguments related to social identity and cultural cognition (Kahan, 2012), it is not obvious which recent changes either within or between the Republican and Democratic ideological camps have created polarized attitudes toward science.

First, we consider the role of the presidency, particularly the efforts taken under the Trump administration to undermine the standing of science in public discourse, and the ability for environmental science to shape public policy (Carter et al., 2019; Frickel and Rea, 2020). While tampering with scientific integrity in policy-making is not unique to the Trump presidency (Berman and Carter, 2018), the effects of past efforts of this kind were limited in terms of their magnitude and duration. They tended to be limited to specific policy domains (e.g. G.W. Bush and climate change), leaving perceptions of general scientific integrity largely intact. Hence, we could expect general trust in science to be resilient against a Trump effect, where any decrease in trust among conservative voters should be similarly limited in scope to specific scientific issues and temporary. Yet, we find a historically unique change in patterns of general scientific trust among Americans emerging at the end of the Trump presidency, suggesting that a distinct phenomenon may be developing. It remains unclear whether these shifting perceptions of science are the evidence of a singular Trump effect, or the product of decades-long processes of undermining scientific trust and its role in public policy as suggested by Gauchat (2023).

Second, applying the emerging theory of individual perceptions of trust as a function of the interplay between an issue’s characteristics and the values embedded in an ideology, one could argue that the widening gap in public trust in science could result from the recent “COVID-19 shock.” Given how public health policies during the pandemic transformed people’s daily lives, pandemic-related changes in attitudes toward medical science could have affected—even dominated—recent perceptions of science. Public health institutions were forced into the political limelight for an extended time, during which trust in public scientific agencies rapidly decreased among conservatives, likely instigated by President Trump’s changing attitudes toward the Centers for Disease Control (CDC) (Hamilton and Safford, 2021). Yet, the pandemic raised the salience of trust in science for all Americans, possibly leading to stronger opinions about the topic (Leonhardt, 2022).

Beyond the pandemic, other issues in recent public discourse could have affected trust dynamics, including debates on education and evolutionary theory (Miller et al., 2022, Nadelson and Hardy, 2015) and transgender rights (Jones et al., 2018; Taylor et al., 2024). On all of these, public authorities and science organizations have drawn on the authority of science in opposition to conservative values and beliefs.

Nevertheless, it remains unclear how theories explaining issue-specific changes in trust in science (ideology–issue interactions) relate to increased partisan polarization of general trust in science as an institution. A long-term trend of ideological divergence of trust in science since the 1990s has been attributed to structural changes within the conservative identity (Gauchat, 2012), driven by macro-social mechanisms such as an increasingly role of expert knowledge and science in governmental decision-making. Oreskes and Conway (2022) argue that general trust in science is “collateral damage” from broader conservative efforts to increase distrust in government. But this also leaves our observations regarding Democrats unexplained. Future research could ask whether and under what conditions issue-specific dynamics, including those related to climate and medical science, can interact and shape general attitudes toward science. Alternatively, one can ask whether the role of science in society as such—the standing and function of the institution—has become a political issue in the same sense that climate change, biofuels, genetically modified organisms (GMOs), or abortion are political issues. That would open “science-in-society” or “science-in-policy making” as a topic to ideological debate and evaluation, and subject to issue-specific trust dynamics.

### Temporary or lasting?

While trust in science in the United States has become increasingly polarized over the last three decades, it is unclear whether the dynamics over the most recent 5 years constitute an aberration or mark the beginning of a more lasting change in the relationship between science, society, and politics. Historically, significant changes in general levels of trust in science never lasted more than 2–3 years, and one could argue that the current situation—while much greater in scope—is likely to be short-lived, too. With fading memories of the pandemic, conservatives’ trust in science might recover, while science could become a less motivating issue for liberals, particularly if the visibility of polarizing political actors fades from the political stage, or liberal political actors with more skeptical attitudes toward science become more salient.

However, the extraordinary recent shifts could indicate that a more profound transition might be taking place. The concept of social tipping points (Otto et al., 2020) can be useful to understand these dynamics. Social tipping refers to non-linear change processes that lead to a fundamental system reorganization (Milkoreit et al., 2018). Stresses can build up gradually in a social system, weakening its resilience over long time periods. However, once the system passes a threshold—the tipping point—self-amplifying feedback dynamics propel the system rapidly toward a new state. The recent widening of the gap in trust between conservatives and liberals appears to fit this pattern. A steady long-term trend since the 1990s indicates the presence of slow processes undermining the system’s resilience, that is, political cohesion, shared political value commitments, and understandings of reality uniting different ideological groups. The size of the shift of trust in science since 2018 can be interpreted as an increase either in the variance or in the rate of change (i.e. acceleration) of the system. Increased variance can be an early warning signal for an impending, but possibly still preventable, tipping point (Bauch et al., 2016; Scheffer, 2009). Or, if these polarizing trends rather signal non-linearity—a speeding up of the system—a tipping threshold might have been passed already. In that case, science–society relationships in the United States might now be moving toward a novel system state, which could be characterized by a permanent divergence in levels of trust in science among Americans. This alternative system would provide a highly unstable context for science engagement in policy and public funding for science.

### Immediate versus long-term implications

What are the implications of the divergence in trust among conservatives and liberals for behavior, policy support, science funding, environmental conditions, public health, and the role of science in society? In the short term, different attitudes toward science can affect a range of individual behaviors and consumption choices of Americans that are shaped by perceptions of science, including environmental behaviors, such as recycling or water conservation, and health-related activities, such as getting a flu vaccine. Less trusting groups would be more likely to disregard scientific advice. Growing mistrust among Republicans might also weaken future policy support for measures in issue domains with a strong reliance on scientific input. Again, environmental and health policy could be most affected, but this is relevant for other domains as well, including education, energy, food, and agriculture. Furthermore, given the differing attitudes among conservatives and liberals, creating political majorities for any policy measure could become more challenging. This might be particularly true for science policy and government funding for science. More long-term implications are hard to assess but the disappearance of a shared appreciation of scientific knowledge as a source of factual information about the world that can serve to identify and solve collective problems could severely undermine societal functioning.

## 5. Conclusion

Our observations indicate that trust in science is moving from a stable background condition of social and political life in the United States—a bedrock of effective collective decision-making—to a widely contested and possibly political issue. Loss of trust in science is no longer a domain-specific issue, such as in the case of climate change. Instead, the United States is witnessing a loss of general trust in science among half the population. Long-term trends of political polarization now include beliefs regarding the trustworthiness of science. This trend is eroding the country’s shared foundation for decision-making, policy, and political discourse—a common understanding of what is real and how reality can be known. Increasingly, ideological groups have come to define themselves and their differences with reference to their belief in science, tying the question of who they are to the question of what is. Given the potentially far-reaching implications of these changes, there is an urgent need to study whether the current trends in public trust in science are of a temporary or more lasting character. Indeed, much of the contemporary challenge of understanding societal transformation dynamics is identifying whether recent observations of increased variance will affect non-linear, tipping dynamics toward a new systemic state (Milkoreit, 2023).

Future empirical work should advance measurement approaches to cultural-institutional perceptions of science. And while we have focused exclusively on the United States, it might be interesting to explore whether similar trust dynamics can be observed in other world regions, for example, related to the growth of right-wing populism in Europe and South America.

## Supplemental Material

sj-docx-1-pus-10.1177_09636625241302970 – Supplemental material for Rapidly diverging public trust in science in the United States Supplemental material, sj-docx-1-pus-10.1177_09636625241302970 for Rapidly diverging public trust in science in the United States by Manjana Milkoreit and E. Keith Smith in Public Understanding of Science
