# Supplementary material for: Rapidly diverging public trust in science in the United States
Source: Public Underst Sci. 2024 Dec 7;34(5):616–27. doi: 10.1177/09636625241302970 (PMC12177194; doi:10.1177/09636625241302970)
Supplement: sj-docx-1-pus-10.1177_09636625241302970 – Supplemental material for Rapidly diverging public trust in science in the United States [file sj-docx-1-pus-10.1177_09636625241302970.docx]

**Rapidly Diverging Public Trust in Science in the United States**

**Online Appendix A**


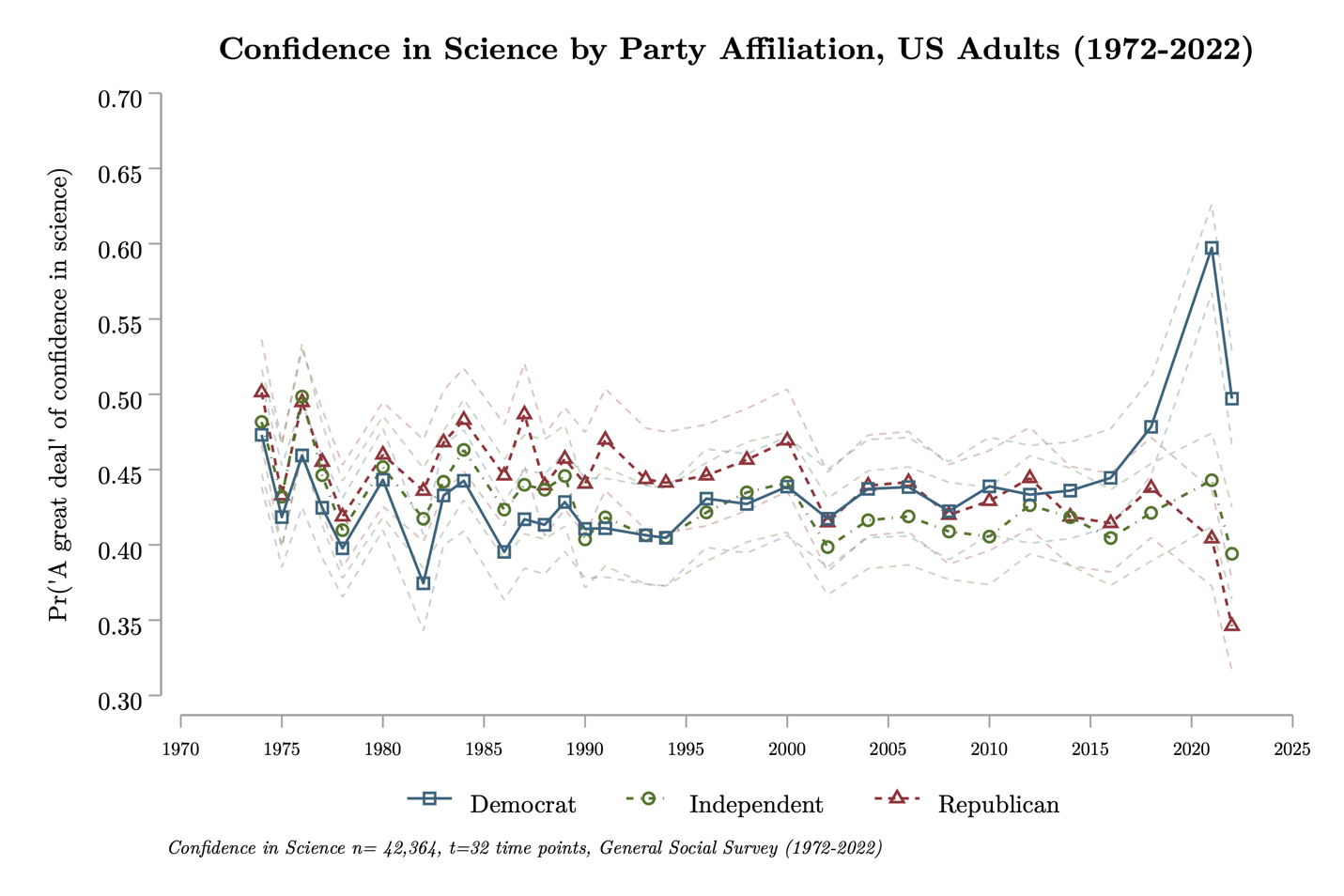


Figure A.1: Percentage of Americans with ‘a great deal’ and ‘hardly any’ confidence in science by party affiliation, 1972-2022. Panel A displayed the percentage of Americans with ‘a great deal’ of confidence in science, from 1972-2022 by party affiliation, while Panel B displays the percentage with ‘hardly any’ confidence in science. Data for Panel A comes from the General Social Survey (1972-2022, n=42,364, t=32 time points).

**
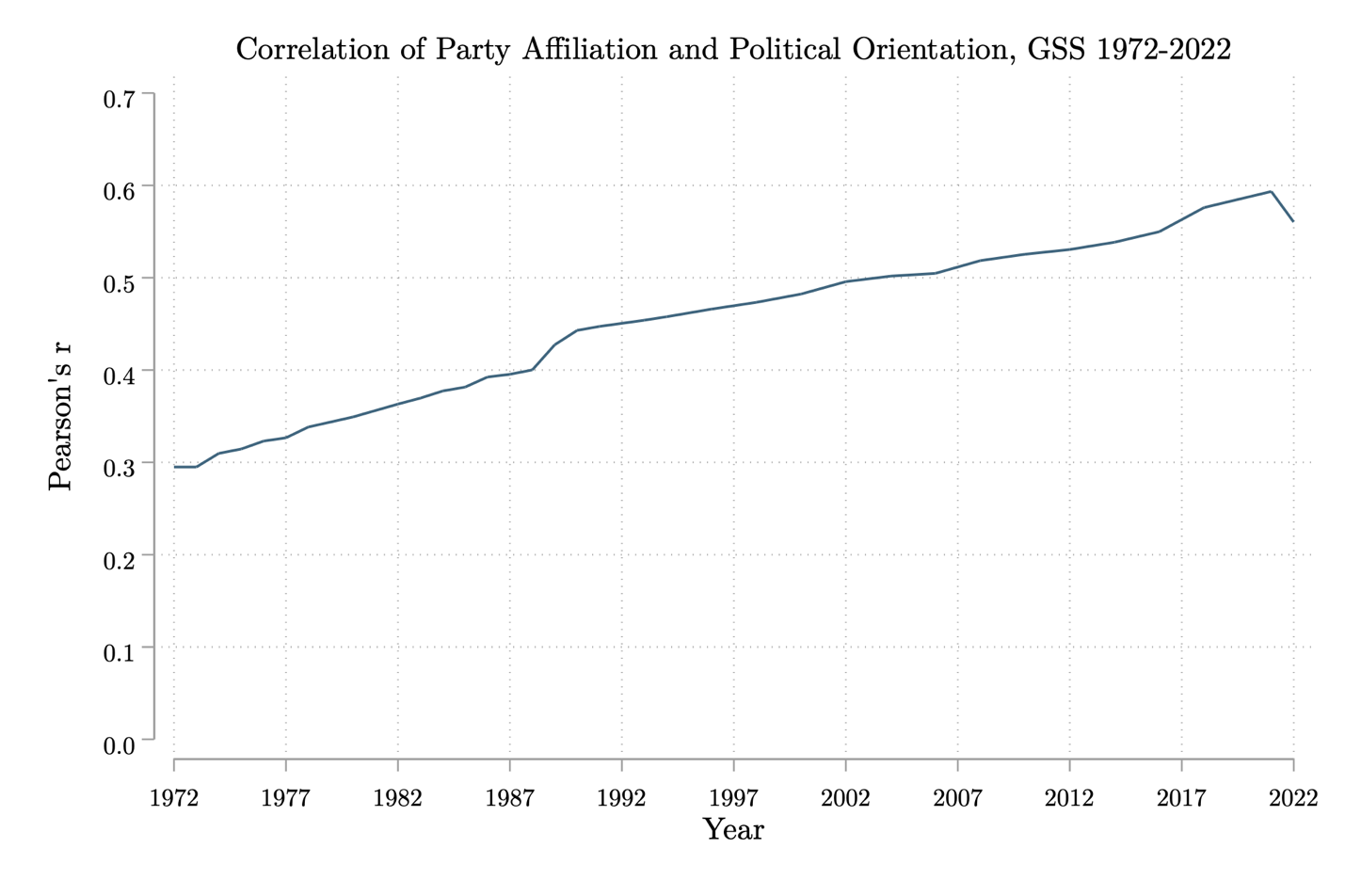
**

Figure A.2: Pearson’s R correlation of association between political orientation and party affiliation, 1972-2022. Data comes from the General Social Survey (1972-2022, n= 42,643, t=32 time points).


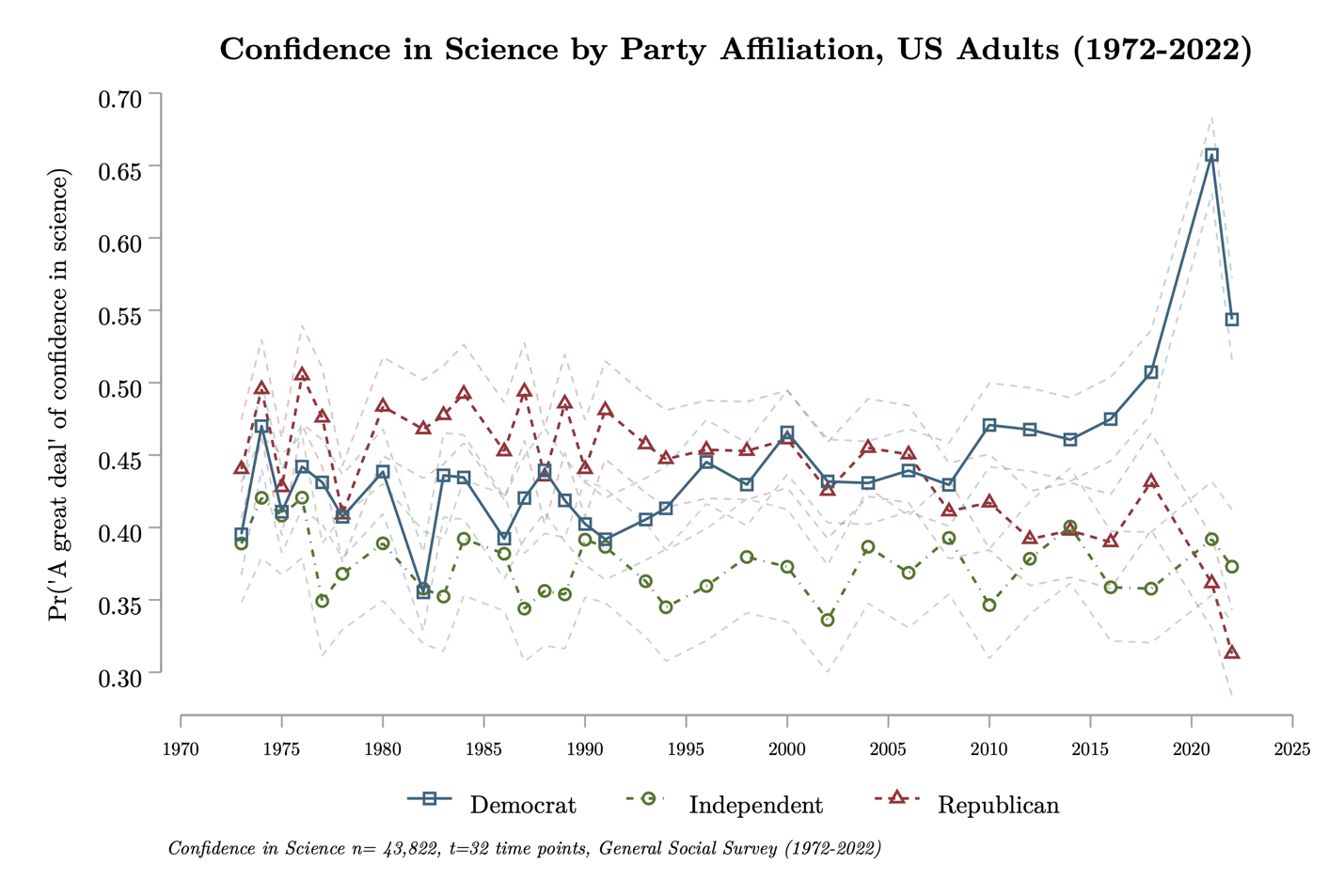


Figure A.3: **The average predicted probability of having ‘a great deal’ of confidence in science, by party affiliation (Democrat, Independent, Republican) in the United States from 1972-2022 including controls for frequency of attendance in religious services.** The average predicted probability is calculated from CCREM estimates of confidence in science by yearly effects, adjusting for the respondent age and birth year cohort, and controlling for gender, ethnic identities, education, and attendance in religious services).  The 95% confidence interval for the average predicted probability is plotted in dashed line.  The data comes from the General Social Survey (1972-2022, n=43,822, t=32 time points).


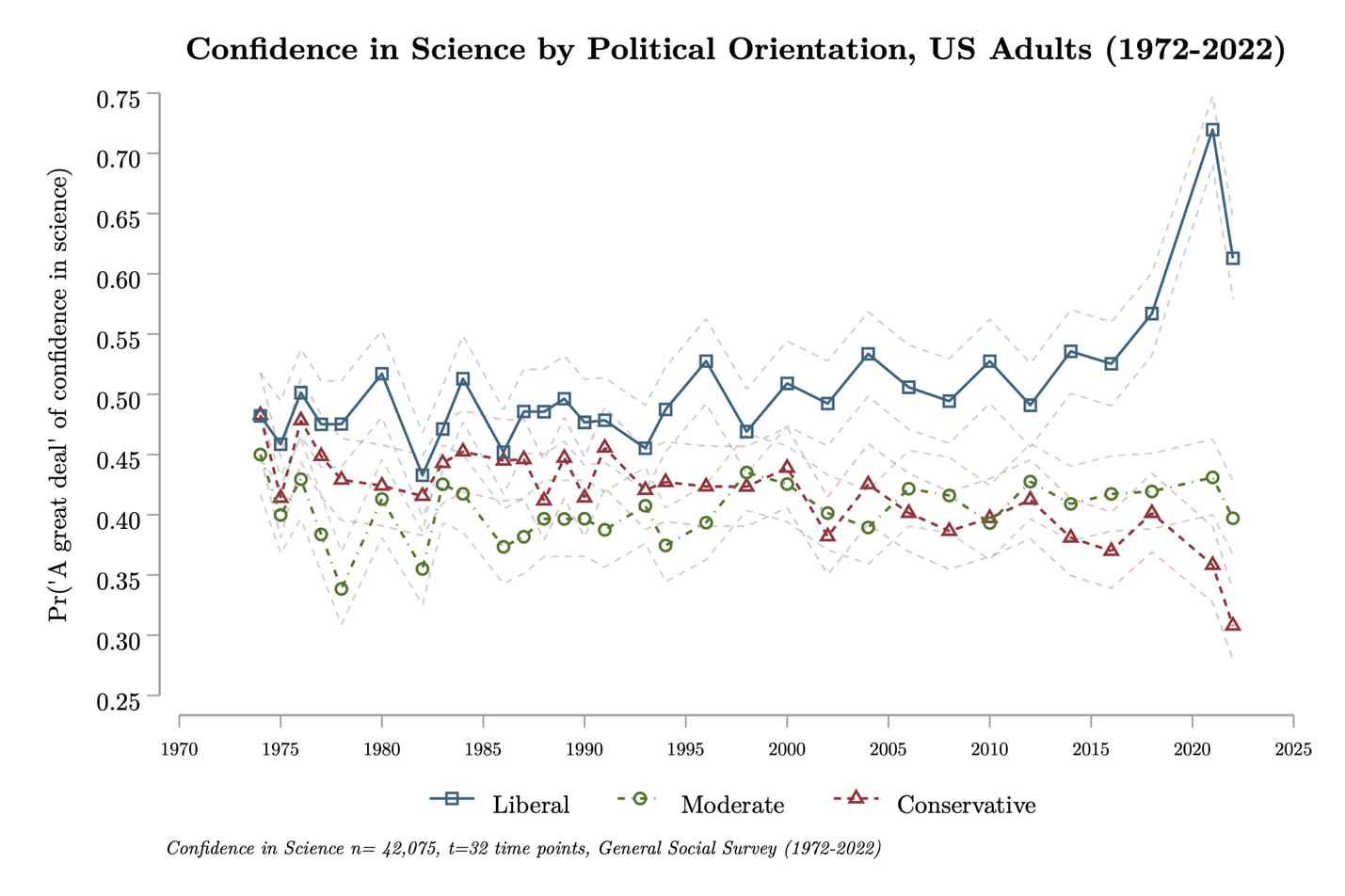
 Figure A.4: **The average predicted probability of having ‘a great deal’ of confidence in science, by political orientation (Liberal, Moderate, Conservative) in the United States from 1972-2022 including controls for frequency of attendance in religious services.** The average predicted probability is calculated from fixed effects estimates of confidence in science by year, adjusting for the respondent age, gender, ethnic identities, education, and attendance in religious services).  The 95% confidence interval for the average predicted probability is plotted in dashed line.  The data comes from the General Social Survey (1972-2022, n=42,075, t=32 time points).


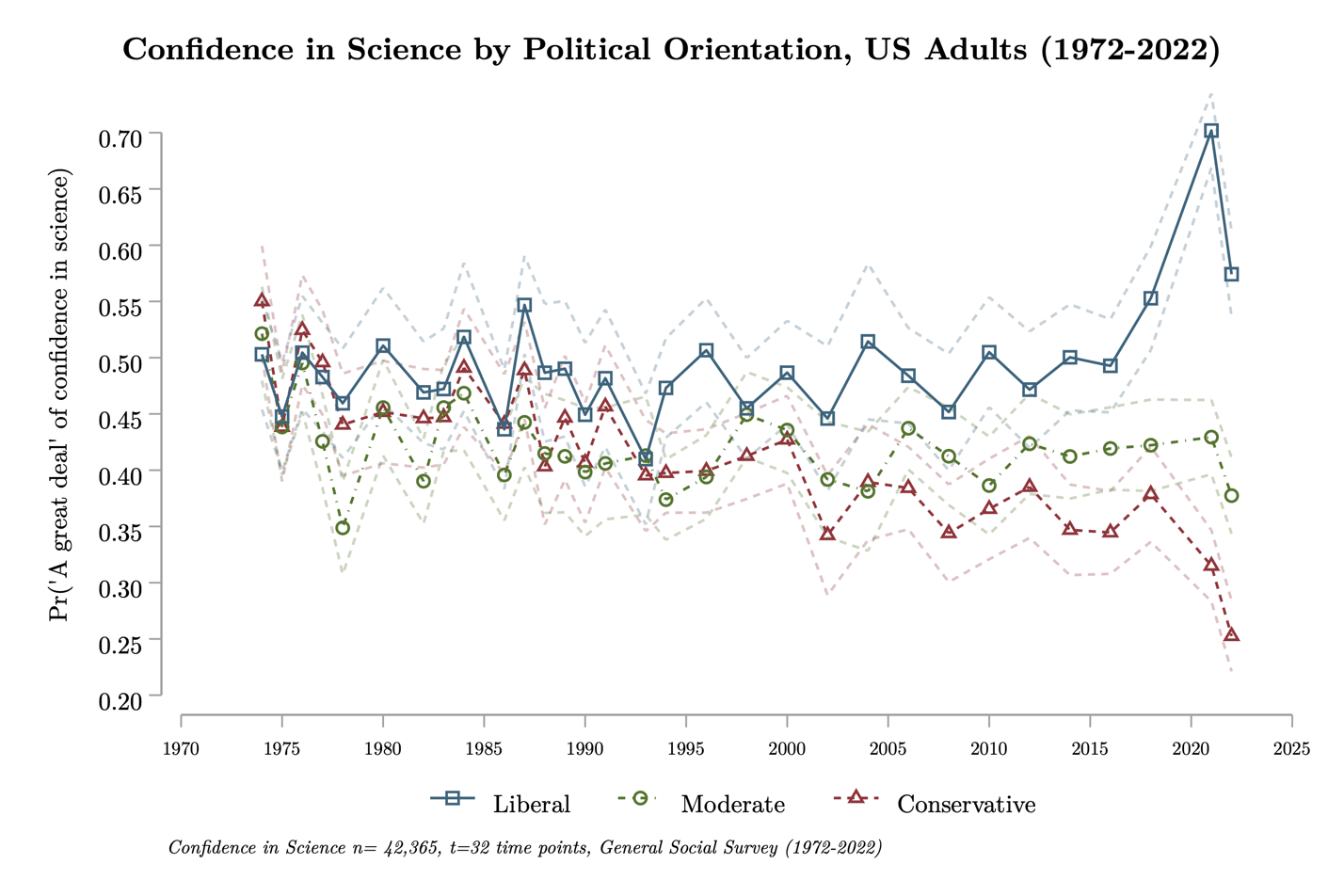


Figure A.5: **The average predicted probability of having ‘a great deal’ of confidence in science, by political orientation (Liberal, Moderate, Conservative) in the United States from 1972-2022 using fixed effects regression estimates.** The average predicted probability is calculated from fixed effects estimates of confidence in science by year, adjusting for the respondent age, gender, ethnic identities, and education).  The 95% confidence interval for the average predicted probability is plotted in dashed line.  The data comes from the General Social Survey (1972-2022, n=42,365, t=32 time points).
